# Stigmatization among Patients with Plaque Psoriasis

**DOI:** 10.3390/jcm12196425

**Published:** 2023-10-09

**Authors:** Barbara Jankowiak, Elżbieta Krajewska-Kułak, Marta Jakoniuk, Dzmitry F. Khvorik

**Affiliations:** 1 Department of Integrated Medical Care, Medical University of Bialystok, 15-096 Białystok, Poland; elzbieta.krajewska-kulak@umb.edu.pl; 2 Department of Invasive Neurology, Medical University of Białystok, 15-096 Białystok, Poland; marta.jakoniuk@umb.edu.pl; 3 Department of Dermatovenerology, Medical University of Grodno, 230009 Grodno, Belarus; chvorik@mail.ru

**Keywords:** psoriasis, stigmatization, psychodermatology

## Abstract

The aim of the study was to analyze the level of stigmatization among patients with plaque psoriasis according to their demographic and clinical characteristics. The study included 122 patients who completed the 6-item and 33-item Feelings of Stigmatization Questionnaire and a sociodemographic survey. The analysis of the 6-item Stigmatization Scale showed a mean stigmatization score of 6.4 points. (Me = 6; s = 3.7); the mean score for the 33-item scale was 81.3 points (Me = 79.5; s = 19.9). Female patients felt stigmatized more often than males. Respondents living in the countryside had a stronger sense of stigmatization in the Sensitivity to Others’ Attitudes (*p* = 0.0238) and Secretiveness (*p* = 0.0234) domains. The presence of psoriatic lesions across the entire body was the only explanatory variable significantly determining the level of stigmatization in the Positive Attitudes domain, either through the main effect or through the interaction with the patient sex. A highly significant difference was found for the feeling of being flawed domain (*p* = 0.044), with a mean score of 13.4 points. The issue of stigmatization in psoriasis deserves more attention, as the analysis of this problem may provide a better insight into the effect of the disease on the patient’s condition, not merely in the context of its clinical manifestation.

## 1. Introduction

Psoriasis is a non-contagious, chronic inflammatory disease of the skin with an immune background. In most patients, the disease remains active for an extended period of time, throughout their entire lifetime. It is estimated that psoriasis affects approximately 1–3% of the general population. While Caucasians are affected more often, psoriasis is rarer in Asian and Western African populations. In Europe, the prevalence of psoriasis is estimated at 2%. The disease occurs in both men and women, regardless of age [1,2].

In plaque psoriasis, inflamed, reddened skin is demarcated well from raised scaly areas, the plaques. Depending on the severity of the disease, the psoriatic lesions may be limited to some areas of the skin or spread across the entire body [3,4,5].

Patients with psoriasis have to cope not only with the skin lesions but also with stress related to the lack of self-acceptance of the disease and the lack of understanding from close relatives and family members. As the largest organ of the human body, the skin plays an essential role in interpersonal contacts, and research showed that dermatological diseases, among them psoriasis, are an important source of social rejection.

The term ‘stigmatization’ refers to a social attitude manifesting as a negative perception and lack of approval for an individual or group. Stigmatization is a consequence of assigning negative roles, values, or labels based on not entirely accurate information, which eventually leads to the discreditation of an individual who is considered impure and alien [6].

Stigmatization is tightly associated with the term ‘stigma’ created by the ancient Greeks to describe marks on the human body representing something evil yet unusual. The marks were cut or burned out, usually for traitors, criminals, or enslaved people. All those people were stigmatized to allow others to avoid them in public [7].

Determinants of stigmatization include sociological factors (discrimination, rejection by a group), stereotypes, disease-related factors (disease manifestations), low education level, self-stigmatization, poor social competencies, and social position [8].

The term ‘stigma’ was introduced to the social sciences in 1963 by Erying Goffman, who identified three types of social stigma [7]:

- physical deformities of the body
- blemishes of individual character perceived as weak will, domineering, or unnatural passions
- the stigma of group identity associated with being of a particular race, religion, nation, etc.

All those types of stigma share some common characteristics, so an individual whom others would otherwise accept becomes ‘different’ and not infrequently ‘worse’ if he/she acquires a visible, repulsive feature. Under such circumstances, other features of such a person are depreciated, even if previously considered positive [7].

Stigmatized patients usually attempt to remove the cause of their stigma. Examples include undergoing rehab in the case of psychoactive substance abuse, cosmetic surgeries to correct physical deformities, and, in the case of patients with dermatological diseases, wearing clothes that cover skin lesions visible to others. Such activities do not make stigmatized persons ‘normal’ but change their identity from those carrying a defect to those who have corrected it.

Unfortunately, no universally accepted intervention exists to attenuate one’s sense of stigmatization. As a result, patients need to learn about their stigma and approve of its social and cultural visualizations, opinions of others, and consequences thereof. Psychological intervention can be helpful [9,10,11], as it may exert a beneficial effect on one’s way of coping with stress, boosting self-esteem, and improving patients’ ability to deal with their stigma. However, not only psychological activities addressed directly to an affected individual play a role in reducing the stigmatization level. Equally important is social support, which may protect one against traumatic experiences associated with being stigmatized. However, social education is believed to play a pivotal role; spreading awareness of psoriasis, with particular emphasis on its non-contagious character, may improve the acceptance of affected patients and promote positive attitudes, as knowledge constitutes a key to reducing social distance [12].

The aim of the study was to analyze the level of stigmatization among patients with plaque psoriasis according to their demographic and clinical characteristics.

The issue of stigmatization in psoriasis deserves more attention, as the analysis of this problem may provide a better insight into the effect of the disease on the patient’s condition, not merely in the context of its clinical manifestation. Our findings and the results published by other authors imply that stigmatization constitutes a significant problem for most patients with psoriasis. Hence, the results of this study add to our knowledge of psychosocial function in patients with dermatological conditions and might stimulate further research in this matter.

## 2. Materials and Methods

### 2.1. Research Material

The study included 122 people. The inclusion criteria of the study were age ≥ 18 years, >1 year elapsed since the diagnosis of psoriasis, Psoriasis Area and Severity Index (PASI) ≤10, and the lack of relevant somatic and mental health disorders within the recent three months.

The respondents were recruited during a hospital stay and/or outpatient visit. The qualification procedure was carried out by experienced dermatologists.

The group included a relatively high proportion of female patients (60%). The mean age was 43.1 years, with a median (Me) of 42. Married persons constituted 62.3%; the vast majority were city dwellers (74.6%). Patients with higher, secondary, and vocational education constituted 40.2%, 39.3%, and 17.2%, respectively; white-collar workers constituted a majority (46.7%).

The average duration of psoriasis was 21 years (Me = 19). Respondents relatively more often declared having a family history of psoriasis (43.7%).

Psoriatic lesions were typically located in the elbows (45.9%), knees (41.8%), head (31.1%), urogenital organs, and buttocks (6.6%), or spread across the entire body (22.1%), back (15.6%), and palms (10.7%). Respondents most often complained about bothersome itchiness (50%); skin scaling was bothersome for up to 34.4% of patients. Redness of the skin was mentioned as a bothersome ailment by 9.8% of patients, and pain/burning sensation by 21.3%. 12.3% of patients reported no psoriasis-related symptoms.

### 2.2. Research Methods

The study patients completed a Polish version of the 6-item and 33-item Feelings of Stigmatization Questionnaire and a survey developed by the authors of this study, containing questions about the sociodemographic characteristics of the participants (gender, age, place of residence, marital status, education, employment status) and information about their disease (location of psoriatic lesions, time elapsed since the diagnosis of psoriasis).

The 6-item scale of stigmatization comprises six single-choice statements. In the Polish adaptation, each answer can score 0 to 3 points, with 0 = never, 1 = sometimes, 2 = very often, and 3 = always. The global score can range from 0 points, interpreted as a lack of stigmatization, to 18 points, which corresponds to maximum stigmatization [13].

The 33-item Feelings of Stigmatization Questionnaire consists of 33 single-choice questions. In the Polish version of the instrument, the answer to each question can be scored on a scale from 0 to 5, where 5 corresponds to “definitely yes”, 4 to “yes”, 3 to “rather yes”, 2 to “rather no”, 1 to “no”, and 0 to “definitely no”. The scale for questions no. 9, 11, 12, 16, 17, 20, 23, 25, and 33 is inverted, so regardless of the question, a higher score corresponds to a higher stigmatization level. The questionnaire is used to determine the level of disease-related stigmatization in six domains: (1) anticipation of rejection, (2) feeling of being flawed, (3) sensitivity to the opinions of others, (4) guilt and shame, (5) positive attitudes, and (6) secretiveness. The overall score of the 33-item Feelings of Stigmatization Questionnaire can range from 0 points (lack of stigmatization) to 165 points (maximum stigmatization level) [13].

Furthermore, a brief validation of both scales was carried out, with the determination of Cronbach’s alpha values. The Cronbach’s alpha for the 6-item scale was 0.849, and even a slightly higher value (0.868) was obtained for the 33-item scale. Both values considerably exceeded the customary cut-off value for Cronbach’s alpha (0.70), confirming the appropriate psychometric characteristics of both scales.

The research conformed with the Good Clinical Practice guidelines, and the procedures followed were in accordance with the Helsinki Declaration.

The study protocol was approved by the Local Bioethical Committee at the Medical University of Bialystok (decision no. APK.002.109.2022).

### 2.3. Statistical Analysis

The significance of differences between two nominal variables was determined with the chi-squared test. The distributions of psychometric measures derived from the 6-item Stigmatization Scale and the 33-item Feelings of Stigmatization Questionnaire are presented with tables along with descriptive statistics for all psychometric variables. The significance of between-group differences in those variables was verified with the Mann-Whitney test. The results were considered statistically significant at *p* < 0.05.

Relationships between pairs of quantitative variables were analyzed based on the Spearman’s coefficients of rank correlation. The power of the relationship was interpreted as follows [14]:

- |*R*| < 0.3—no correlation;
- 0.3 ≤ |*R*| < 0.5—weak correlation;
- 0.5 ≤ |*R*| < 0.7—moderate correlation;
- 0.7 ≤ |*R*| < 0.9—strong correlation;
- 0.9 ≤ |*R*| < 1—very strong correlation;
- |*R*| = 1—ideal correlation.

Additionally, the test for the significance of correlation coefficients (*p*) was conducted to verify whether the relationship found in the sample reflected the association in the general population or was random. The results were considered significant at *p* < 0.05.

Demographic, social, and clinical determinants of stigmatization measures were determined based on regression analysis. The analysis was based on linear regression models containing the patient’s sex, place of residence, age, duration of psoriasis, location of psoriatic lesions in the scalp or face, and spreading psoriatic lesions throughout the entire body as potential independent determinants of stigmatization levels. We assumed that spreading psoriatic lesions across the entire body or the location thereof in the scalp or face (exposed body parts) may determine the level of stigmatization. Each psychometric variable was analyzed individually as a dependent variable. Moreover, progressive stepwise regression analysis was carried out to identify factors having a significant effect on each dependent variable.

As preliminary analysis demonstrated a strong effect of patient sex on the values of stigmatization measures, the regression models also included second-degree interactions between sex and other potential determinants.

The statistical analysis was carried out with the STATISTICA 12.5 package.

## 3. Results

The analysis of the distribution of psychometric measures derived from the 6-item Stigmatization Scale showed that mean scores for respondents were 6.4 points (Me = 6; s = 3.7). On the other hand, the analysis of the distribution of psychometric measures from the 33-item Stigmatization Scale showed that the average rating of the respondents was 81.3 points (Me = 79.5; s = 19.9).

Female patients felt stigmatized more often than males. Statistically significant differences between women and men were observed in the case of nearly all overall and specific measures derived from the 6-item Stigmatization Scale and the 33-item Feelings of Stigmatization Questionnaire (Table 1).

Patient age, age at the diagnosis of psoriasis, and duration of the disease did not influence the sense of stigmatization (Table 2).

Patient education (vocational vs. secondary vs. higher) did not exert a significant effect on the sense of stigmatization. The *p*-values for the overall scores of the 6-item Stigmatization Scale and the 33-item Feelings of Stigmatization Questionnaire were 0.1652 and 0.7872, respectively.

Respondents living in the countryside had a stronger sense of stigmatization, as shown by the overall scores for the 6-item Stigmatization Scale and the 33-item Feelings of Stigmatization Questionnaire and scores for some specific domains of the latter. Specifically, respondents living in the countryside experienced a stronger sense of stigmatization in the Sensitivity to Others’ Attitudes (*p* = 0.0238) and Secretiveness (*p* = 0.0234) domains (Table 3).

Family history of psoriasis had no significant effect on the sense of stigmatization, with the *p*-values for the 6-item Stigmatization Scale and the 33-item Feelings of Stigmatization Questionnaire scores equal to 0.3108 and 0.7629, respectively.

### Regression Models for Stigmatization

Two statistically significant determinants, sex, and place of residence, were identified in the regression models, with the overall scores for the 6-item Stigmatization Scale and the 33-item Feelings of Stigmatization Questionnaire as the outcome variables. The goodness of fit of both models was similar. Based on the regression coefficient (*B*) values, male patients appeared to experience lower levels of stigmatization than females (by 1.85 points on average for the 1-item Stigmatization Scale (Table 4) and by 10.71 points on average for the 33-item Feelings of Stigmatization Questionnaire (Table 5)). Respondents living in the countryside presented with higher levels of stigmatization than city-dwellers (by 1.95 points and 10.70 points on average for the 6-item Stigmatization Scale and the 33-item Feelings of Stigmatization Questionnaire, respectively).

Sex turned out to be the only determinant of stigmatization in the Feeling of Being Flawed domain, with the mean scores for women being 2.25 points higher than for men (*p* = 0.0172).

The results of the regression analysis for the Sensitivity to Others’ Attitudes domain were similar to the overall scores: females experience higher levels of stigmatization than males (*p* = 0.0023) and respondents living in the countryside (*p* = 0.048).

However, different results were obtained for the Positive Attitudes domain of the 33-item Feelings of Stigmatization Questionnaire (Table 6). The regression model for this outcome variable was the only one to include a significant clinical determinant, the location of psoriatic lesions. The presence of the lesions across the entire body was the only explanatory variable determining the stigmatization level in that domain, both through the main effect and the interaction effect with patient sex.

The results of the regression analysis for the last specific domain of the 33-item Feelings of Stigmatization Questionnaire, Secretiveness, were similar to the overall scores, with lower stigmatization levels observed in men (0.0022) and city-dwellers (*p* = 0.0007).

## 4. Limitation

The analysis study did not include a relationship between PASI and stigmatization, as only patients with PASI ≤ 10 and moderately severe psoriasis were enrolled, which is a limitation of this study.

## 5. Discussion

The problem of stigmatization among patients with skin diseases is often addressed in psychodermatology research [9,15,16]. Psoriasis, one of the most common dermatoses, particularly predisposes to stigmatization in the affected patients [10,11,12,17,18].

Due to the ‘defects’ in their appearance, patients with psoriasis often experience negative attitudes and responses from others, such as disgust or reluctance, critical comments, and avoidance of contact. Under such circumstances, stigmatization has an unfavorable effect on self-acceptance and self-esteem, may deteriorate the quality of life, and also impair the social functioning of the patients [19]. Additionally, emotions such as anxiety, depressed mood, and stress may eventually lead to depression. Stigmatization is considered a stressor that activates the disease and triggers the development of new skin lesions, which further deteriorates the psychological condition of the patients (deteriorated emotional status results in the release of proinflammatory cytokines, which in turn has a detrimental effect on skin function).

One of the determinants of stigmatization analyzed in the present study was patient sex. Under social standards, women are evaluated based on their appearance more frequently than men [20,21], and psoriasis is a condition with a profound effect on appearance. It needs to be emphasized that this tendency was also observed in our present study, albeit solely. The over-the-33-item Feelings of Stigmatization Questionnaire was approximately 10 points higher among women than in men (mean 85.0 points vs. 75.6 points, *p* = 0.0129).

The results of other studies on this matter are inconclusive. In some studies, patient sex was associated with the stigmatization level, with women feeling more stigmatized than men [17,22,23,24,25]. Meanwhile, in other studies, male patients presented with higher stigmatization levels [14,26,27,28,29,30,31], or no association was found between patient sex and psychological condition [32,33].

Another factor analyzed in the present study was the place of residence. Our analysis showed that people living in the countryside presented with higher stigmatization levels than other respondents. This observation might be associated with the fact that patients who live in the countryside have less anonymity, whereas those living in larger agglomerations can remain ‘unnoticed’ by others. A higher level of stigmatization among patients living in the countryside was also documented in one previous study [27].

The location of psoriatic lesions is important for the psychosocial functioning of the patient. The presence of lesions on exposed body parts is known to be associated with higher levels of anxiety and stress and evokes the sense of ‘being different’ [34]. The characteristic reddened areas of the skin covered with silverish scales can be considered by others as ‘impure’ and/or contagious, which evokes adverse social reactions with the avoidance of physical contact, e.g., shaking hands [35,36]. The patients themselves also tend to limit their social interactions, avoid swimming and sunbathing, use public transformation, visit hairdressers, etc. [37,38,39,40,41].

The present study also showed an interaction between patient sex and the location of psoriatic lesions. Having psoriatic lesions in the areas that could not be covered easily, such as the scalp and face, women presented with higher stigmatization levels than men. Additionally, the location of psoriatic lesions exerted an effect on the scores for the Positive Attitudes domain. Regression analysis demonstrated that spreading psoriatic lesions across the entire body was associated with an increase in the stigmatization level in this domain by approximately 1.39 points on average.

In the study by Hawro et al., higher levels of stigmatization were also observed among patients who declared that they could not cover their skin lesions (*p* = 0.025) [42]. Similar findings were also reported by other authors, who found a link between the distribution of psoriatic lesions throughout the body and stigmatization level [16,42,43,44]. However, according to Hrehorów et al. [12], the presence of psoriatic lesions on the face did not change the level of stigmatization. Meanwhile, the occurrence of psoriatic lesions in this location was the strongest determinant of stigmatization, according to Dimitov et al. [45].

Our study showed no association between education level and the sense of stigmatization. However, according to Lu et al., worse education is a determinant of stigmatization [11]. A similar relationship was also reported by van Beugen et al. (*p* = 0.01) [44].

According to some researchers, higher stigmatization levels were observed among older people [23], whereas others found stigmatization to be higher in younger patients [11,26]. In our present study, age exerted no effect on the stigmatization level.

Studies analyzing the effects of various factors on the quality of life in psoriasis have been conducted for years [38,46,47,48,49,50]. Psychological condition and social position play important roles in psoriasis, as those factors can potentially trigger or exacerbate the disease. In turn, stigmatization is highlighted as a strong determinant of psychological condition and, as such, can have a detrimental effect on the quality of life [26,27].

The observation mentioned above was confirmed in our present study. The respondents who confirmed that psoriasis had an effect on their quality of life presented with higher stigmatization levels than other participants. The analysis of the results obtained with the 6-item Stigmatization Scale showed that moderate to high levels of stigmatization were experienced by 49.2% of patients. Low levels of stigmatization were found in 50.8% of patients.

According to van Beugen et al., up to 73% of patients with psoriasis experienced stigmatization [44], consistent with results published by other authors [12,31,33].

Accurate determination of stigmatization level can constitute a challenge, as this parameter is determined by various qualitative and quantitative factors, some of which change over time.

## 6. Conclusions

Women and respondents living in the countryside present higher levels of stigmatization due to psoriasis. The location of psoriatic lesions is important for the psychosocial functioning of the patient. Patient age, age at the diagnosis of psoriasis, duration of the disease, family history of psoriasis, and education did not influence the stigmatization level.

## Figures and Tables

**Table 1:** Relationships between stigmatization levels and patient’s sex.

Stigmatization Scale	Women			Men			*p*
	$\bar{x}$	Me	*s*	$\bar{x}$	Me	*s*	
Stigmatization Scale (6-Item) Summary measure	7.0	7.5	3.2	5.4	5	4.2	0.0147
Stigmatization Scale (33-Item) Summary measure	85.0	84.5	19.2	75.6	75.5	19.8	0.0129
-Anticipation of rejection	23.5	24	6.3	21.3	22	6.5	0.0921
-Feeling of being flawed	14.3	14	5.1	12.0	11.5	4.8	0.0095
-Sensitivity to the opinions of others	12.7	13	3.8	10.7	10.5	4.3	0.0129
-Guilt and shame	14.0	14	3.5	14.3	14	3.2	0.7720
-Positive attitudes	9.0	9	2.6	7.8	8	3.0	0.0275
-Secretiveness	11.4	12	3.5	9.6	9	4.3	0.0044

*p*—*p*-value for the Mann-Whitney test; $\bar{x}$—arithmetic mean; Me—median; *s*—standard deviation.

**Table 2:** Correlation of stigmatization with patient’s age, age at the diagnosis of psoriasis and duration of the disease.

Stigmatization Scale	Patient’s Age	Age at the Diagnosis	Duration of the Disease
Stigmatization Scale (6-Item) Summary measure	−0.15 (0.1058)	−0.04 (0.6371)	−0.08 (0.3760)
Stigmatization Scale (33-Item) Summary measure	−0.10 (0.2697)	−0.10 (0.2634)	−0.01 (0.8828)
-Anticipation of rejection	−0.07 (0.4479)	−0.12 (0.2062)	0.04 (0.6962)
-Feeling of being flawed	−0.07 (0.4185)	−0.07 (0.4729)	−0.03 (0.7757)
-Sensitivity to the opinions of others	−0.13 (0.1659)	−0.08 (0.3949)	−0.06 (0.5457)
-Guilt and shame	0.03 (0.7049)	−0.04 (0.6569)	0.07 (0.4201)
-Positive attitudes	0.02 (0.7871)	−0.01 (0.9544)	0.03 (0.7758)
-Secretiveness	−0.14 (0.1155)	−0.12 (0.1818)	−0.04 (0.6822)

**Table 3:** Relationships between stigmatization and place of residence.

Stigmatization Scale	Countryside			City			*p*
	$\bar{x}$	Me	*s*	$\bar{x}$	Me	*s*	
Stigmatization Scale (6-Item) Summary measure	7.6	8	4.1	6.0	5	3.5	0.0439
Stigmatization Scale (33-Item) Summary measure	87.8	87	18.0	79.1	77	20.1	0.0353
-Anticipation of rejection	23.9	23	6.5	22.2	22	6.4	0.2409
-Feeling of being flawed	14.4	14	4.5	13.1	13	5.3	0.2480
-Sensitivity to the opinions of others	13.4	14	3.3	11.5	12	4.3	0.0238
-Guilt and shame	15.0	15	3.4	13.8	14	3.3	0.1358
-Positive attitudes	8.7	9	2.5	8.5	8	3.0	0.6397
-Secretiveness	12.5	12	4.1	10.1	10	3.8	0.0234

**Table 4:** Coefficients of regression for sex and place of residence (6-Item).

Independent Predictors	Stigmatization Measure (6-Item) *R*^2^ = 9.3%, *F* = 6.0, *p* = 0.0032		
	*B* (95% c.i.)	*p*	*β*
sex (male vs. female)	−1.846 (−3.174; −0.517)	0.0069	−0.25
place of residence (countryside vs. city)	1.954 (0.471; 3.438)	0.0103	0.23

Overall statistics: *R*^2^—coefficient of determination (the percentage of the response variable variation that is explained by a model); Test statistic *F* and *p*-value for assessment of significance of whole model. Results for each predictor variables: *B*—regression coefficient (with 95% confidence interval); *p*—assessment of significance; *β*—standardize regression coefficient.

**Table 5:** Coefficients of regression for sex and place of residence (33-Item).

Independent Predictors	Stigmatization Measure (33-Item) *R*^2^ = 10.2%, *F* = 6.7, *p* = 0.0018		
	*B* (95% c.i.)	*p*	*β*
sex (male vs. female)	−10.713 (−17.844; −3.581)	0.0036	−0.26
place of residence (countryside vs. city)	10.700 (2.737; 18.663)	0.0089	0.24

**Table 6:** Coefficients of regression for the Positive Attitudes domain.

Independent Predictors	Positive Attitudes (33-Item) *R*^2^ = 9.3%, *F* = 6.0, *p* = 0.0032		
	*B* (95% c.i.)	*p*	*β*
entire body	1.387 (0.183; 2.590)	0.0243	0.20
entire body × sex (male vs. female)	1.534 (0.526; 2.542)	0.0032	0.27

## Data Availability

Data are available upon reasonable request.
